# Clinical trajectories of patients with multiple sclerosis from onset and their relationship with serum neurofilament light chain levels

**DOI:** 10.3389/fneur.2024.1477335

**Published:** 2024-10-30

**Authors:** Carlos Quintanilla-Bordás, Laura Cubas-Núñez, Jéssica Castillo-Villalba, Sara Carratalá-Boscá, Raquel Gasque-Rubio, Jordi Tortosa-Carreres, Carmen Alcalá, Lorena Forés-Toribio, Celia Lucas, David Gorriz, Francisco Pérez-Miralles, Bonaventura Casanova

**Affiliations:** ^1^Neuroimmunology Research Group, Health Research Institute La Fe, Valencia, Spain; ^2^Laboratory Department, University and Polytechnic Hospital La Fe, Valencia, Spain; ^3^Systems and Applications Engineer Department, University and Polytechnic Hospital La Fe, Valencia, Spain

**Keywords:** multiple sclerosis, neurofilament light chain, progression, relapses, biomarkers

## Abstract

**Background:**

Serum neurofilament light chain (sNfL) is a biomarker of neuroaxonal destruction that correlates with acute inflammation (AI) in multiple sclerosis (MS). However, in the treatment era, progression without AI is the main driver of long-term disability. sNfL may provide added value in detecting ongoing axonal damage and neurological worsening in patients without AI. We conducted a prospective three-year study on patients with a first MS relapse to evaluate the basal cut-off value predicting early increased disability unrelated to relapses.

**Methods:**

sNfL levels and AI presence were measured every 6 months during the first year and the Expanded Disability Status Scale (EDSS) was monitored until the third year. Baseline cohorts were stratified by sNfL levels, using a cut-off derived from patients without AI (absence of clinical relapses, new/enlarging T2 lesions, or gadolinium enhancement in magnetic resonance imaging) at year one.

**Results:**

Fifty-one patients were included. A sNfL cut-off of 11 pg/mL predicted sustained neurological worsening independent of AI. Patients exceeding this threshold exhibited features of highly active MS (higher proportion of AI, oligoclonal M bands and higher EDSS). Despite AI ablation, sNfL levels persisted elevated and were significantly associated with increased EDSS at baseline and year 3. Patients with low sNfL and concurrent AI (*n* = 8) experienced relapses in the optic nerve, brainstem, and spinal cord topographies.

**Conclusion:**

sNfL elevation may detect patients with increased disability even when AI is controlled. This may reveal mechanisms associated with early axonal degeneration and help identify patients at higher risk of progression.

## Introduction

Multiple sclerosis (MS) is characterized by two phenomena: (1) acute inflammation (AI), defined by the presence of clinical relapses, new or enlarging T2 lesions, or gadolinium enhancement in magnetic resonance imaging (MRI), that is mainly responsible for relapse-associated worsening (RAW); and (2) chronic inflammation, associated to neurological worsening in absence of AI (1). The relative contribution of these phenomena shape a continuum of clinical phenotypes that move beyond the classical distinction between relapsing–remitting (RRMS), primary or secondary progressive MS (PPMS and SPMS) (2). Recent efforts have been made to detect progression in patients previously considered inactive. In this regard, progression independent of relapse activity (PIRA) and MRI activity (PIRMA) have emerged as concepts to identify patients ongoing progression outside of clinical relapses (3). In fact, in the actual treatment era, progression in the absence of AI is the main driver of disability accumulation, especially at later stages (4). Chronic inflammation arising from smoldering lesions, subpial cortical demyelination and meningeal lymphoid aggregates has been mechanistically related to this concept. However, they cannot be readily detected by conventional MRI studies (5). Currently, several soluble biomarkers have been investigated to detect PIRA in MS, especially neurofilament light chain (NfL), a biomarker of neuroaxonal destruction (6–8), and glial fibrillary acidic protein (GFAP), a biomarker of glial activation (9).

Serum NfL (sNfL) has shown to correlate with AI in the form of clinical relapses or radiological activity (10). Benkert et al. also showed that patients with sNfL levels above 10 pg/mL predicted poor prognosis (11). This brought considerable interest, as sNfL could be monitored in clinical practice. Still, the value of sNfL levels in patients that do not exhibit signs of AI and its potential to detect chronic and persistent inflammation associated with neurological worsening in these patients has not been fully elucidated. In the aforementioned study, sNfL of patients meeting NEDA-3 status (no increase in EDSS and no new or enlarging T2 lesions or gadolinium-enhancing lesions) prognosticated future disease activity. With respect to EDSS, other studies showed an association between NfL elevation, EDSS progression and PIRA in several cohorts that were followed up for 12 months (12, 13). However, newer studies have challenged this observation: a recent study of patients in whose AI was ablated with anti-CD20 therapies, GFAP but not sNfL predicted confirmed disability worsening (CDW) (14). In another study, high baseline sNfL levels predicted new T2 lesions, clinical relapses but not EDSS progression after 2 years (15), which is also in line with other studies of different time frames (16, 17).

A previous work by our group showed that patients presenting oligoclonal M bands (OCMB) in cerebrospinal fluid (CSF) – a known biomarker of poor prognosis - had sNfL levels above 10 pg/mL, despite not having signs of AI. We suggested that the presence of OCMB might unveil persistent, yet subclinical, inflammation not detected by conventional MRI or by the presence of relapses (18). Similarly, patients with no evidence of AI, who did not show neurological worsening, thereby meeting NEDA-3 criteria (no increase in EDSS and no new or enlarging T2 lesions or gadolinium-enhancing lesions), have shown to have mean sNfL levels below 10 pg/mL. Together, the data suggest that high sNfL levels in patients without AI may disclose chronic inflammation associated with poor prognosis. The objective of this work is to explore the prognostic utility of sNfL levels in determining the clinical trajectories of patients with and without signs of acute inflammation (AI).

## Methods

We included consecutive patients referred to our center experiencing a first relapse suggestive of MS that fulfilled 2017 McDonald criteria (19) with a follow-up of 3 years. We determined the sNfL threshold associated with short-term prognosis in patients not exhibiting signs of AI and then we applied it to stratify the cohorts.

The sNfL threshold was applied retrospectively to define, at baseline, the cohorts presenting with high and low sNfL levels (h-sNfL and l-sNfL, respectively). The cohorts were further classified according to the presence of AI at baseline, month 6 and 12. EDSS scores at baseline, months 6, 12 and 36 were collected.

EDSS outcomes at month 36 of h-sNfL and l-sNfL cohorts were compared. We also analyzed the effect of having AI during the first year (at months 6 and 12), as well as the impact of disease-modifying therapy (DMT) usage [high efficacy therapies (HETs) versus moderate efficacy therapies (METs)].

AI was defined by the presence of clinical relapses, enlarging or new T2 lesions, or gadolinium enhancement on an MRI performed within 90 days. At baseline, enlarging or new T2 lesions were excluded to this definition as no previous MRI were available. Clinical visits were scheduled every 3 months. sNfL levels were determined at baseline, month 6 and 12. EDSS were assessed at each clinical visit throughout the 3-year follow-up period. Brain and cervical spinal cord MRI scans were performed at baseline, and then, annually, or earlier at the discretion of the neurologist. DMT was initiated after clinical disease onset as per clinical practice. Generally, HETs were initiated in patients with aggressive MS at presentation, while METs were initiated in the rest of the cases. Aggressive MS was considered in cases presenting with spinal cord lesions, OCMB in the CSF, 20 or more T2 lesions, 2 or more gadolinium-enhancing lesions at disease onset, 2 relapses within 1 year or incomplete recovery from a relapse. Natalizumab, anti-CD20 monoclonal antibodies, alemtuzumab and autologous stem cell transplant were considered as HETs. Teriflunomide, dimethyl fumarate, fingolimod and cladribine were considered as METs. Patients under MET with breakthrough disease are generally switched to HET.

### Ethics approval

The study was approved by the ethics committee at University and Polytechnic La Fe Hospital of Valencia, Spain, and was therefore performed in accordance with the ethical standards laid down in the 1964 Declaration of Helsinki and its later amendments.

### sNfL determination

sNfL levels were determined with ultrasensitive Simoa™ platform, using Simoa® NF-Light™ V2 Advantage kit (Ref#: 104073) for Simoa™ SR-X Analyzer® instrument (Quanterix Corporation, Boston, USA), according to the manufacturer instructions (20). The functional lower limit of quantification for the NfL concentration was 2.56 pg/mL. Two types of quality-control samples provided in the kit were measured in each plate in duplicate, ensuring measurement validity. All measured values were within the calibration range. The mean intra-assay and inter-assay CVs were less than 10%. All coefficients of variation of concentrations of duplicate determinations were less than 20%.

### MRI studies

MRIs were performed on a 3 T Philips Achieva scanner with standard head coil. EM protocol included axial 3D T1 (echo time 3 ms; repetition time 8 ms; slice thickness 1 mm) and sagittal 3D T2-FLAIR (echo time 2.6 ms; repetition time 6,000 ms; slice thickness 1 mm) brain sequences. MRIs were processed using different specialized software programs. Brain atrophy was quantified using *Freesurfer* software version 5.3 for volumetric image analysis; T2 lesion volume was quantified using the *Lesion Segmentation Toolbox* for Statistical Parametric Mapping; and spinal cord atrophy was measured using ITK-SNAP and the technique of five consecutive slices mean area (21).

### Statistical analysis

To calculate the optimal sNfL threshold associated with increased disability in patients without AI, we conducted a receiver operating characteristic curve (ROC) to classify patients reaching an Expanded Disability Status Scale (EDSS) of 2.0 or more by the end of the follow-up. Such EDSS was used because the study population had very early MS (i.e., first relapse), and previous observations show that reaching a sustained EDSS of 2.0 as a sequel after the first relapse correlates with future progression (22). Since the presence of AI is known to influence sNfL, we used sNfL levels of patients who did not exhibit signs of AI by the end of 1 year of follow-up. A multivariable Random Forest analysis was performed to assess the impact of some variables on the classification of the high-sNfL and low-sNfL cohorts (Supplementary Figure 2).

Normality of variables were assessed using Kolmogorov–Smirnov test. Student’s T or Mann–Whitney U test were used for comparisons for normal and non-normal continuous variables, respectively. Continuous variables are presented as mean ± standard deviation (SD) for normally distributed data and as median with interquartile range (IQR) for non-normally distributed data. Categorical variables are summarized as frequencies and percentages. Correlations were assessed using Spearman’s rank correlation test. Statistical significance was considered for *p*-value <0.05.

## Results

### Study population

**Figure 1:**
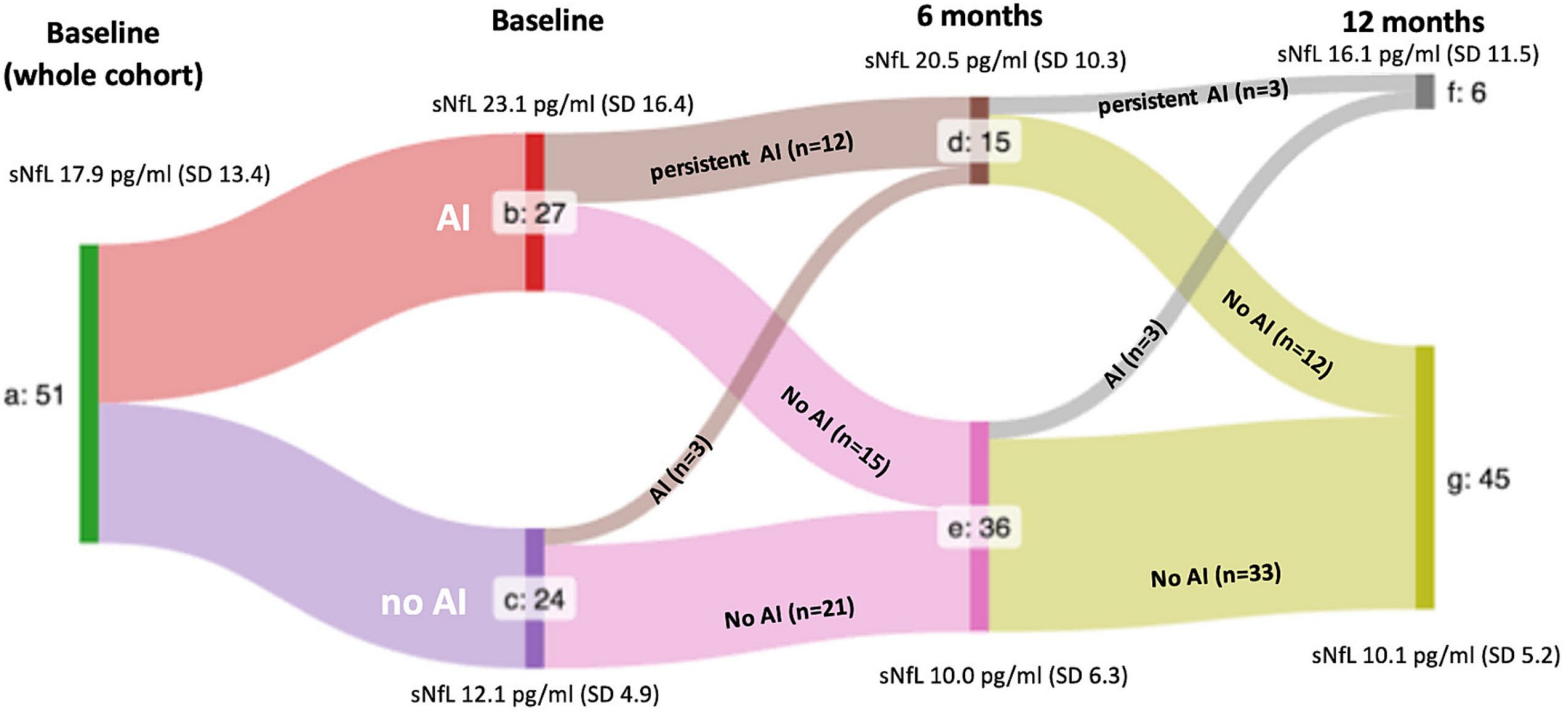
Flowchart of serum neurofilament light chain levels in the whole cohort stratified upon the presence of inflammatory activity at each time point. a: whole cohort. b, d, and f: patients presenting signs of acute inflammation (AI), c, e, and g: patients without signs of AI. Mean sNfL levels are shown. SD: standard deviation. *p*-values after stratification by the presence of AI were 0.003 at baseline, 0.017 at month 6 and 0.03 at month 12.

59 out of 68 patients evaluated with a first episode suggestive of MS met the 2017 McDonald criteria. Of these, 54 patients were diagnosed with relapsing–remitting MS, but 3 were excluded; two due to incomplete follow-up and another one that refused standard of care treatment. Hence, 51 patients were finally included. All patients initiated DMT. Clinical and demographic characteristics of the cohorts are shown in Table 1. Figure 1 shows a flow diagram depicting sNfL levels of patients based on the presence of acute inflammation (AI) at baseline, month 6, and month 12.

**Table 1:** Clinical and demographic characteristics of the cohorts.

	Total series	Low-sNfL cohort	High-sNfL cohort	*p*-value
*n*	51	18	33	
Females, *n* (%)	38 (74.5)	10 (55.6)	28 (84.8)	0.022
Age at symptom onset (mean, SD)	36.3 (11.6)	37.3 (9.5)	35.6 (12.8)	n.s.
Months from symptom onset to sNfL determination (mean, SD)	5.2 (8.1)	4.8 (8.1)	5.4 (8.2)	n.s.
sNfL level in pg/mL (mean, SD)				
Baseline (total)	17.9 (13.4)	7.3 (1.8)	23.7 (13.5)	<0.001
Patients without AI	12.1 (4.9)	7.9 (1.8)	15.1 (4.1)	
Patients with AI	23.1 (16.4)	6.6 (1.7)	30.0 (14.7)	
At month 6 (total)	11.9 (8.2)	9.5 (7.8)	13.5 (8.4)	n.s.
Patients without AI	10.0 (6.3)	7.0 (3.1)	11.9 (7.2)	
Patients with AI	20.5 (10.3)	27.1 (9.5)	18.6 (10.4)	
At month 12 (total)	10.8 (6.3)	7.9 (3.4)	12.5 (7.1)	0.014
Patients without AI	10.1 (5.2)	7.6 (3.3)	11.4 (7.6)	
Patients with AI	16.1 (11.5)	9.3 (7.6)	22.8 (13.5)	
EDSS (median, IQR)				
At baseline	2.0 (1.5)	1.0 (1.0)	2.0 (1.7)	0.002
At month 12	1.5 (1.5)	1.0 (1.0)	2.0 (2.5)	0.049
At month 36	1.5 (1.5)	1.0 (0.9)	2.0 (2.5)	0.013
Disease evolution time from symptom onset in years (mean, SD)	2.6 (0.9)	2.7 (0.9)	2.5 (1.0)	n.s.
Presence of OCGB, *n* = 49 (%)	42 (85.7)	13 (76.5)	29 (90.6)	n.s.
Presence of OCMB, in CSF, *n* = 44 (%)	23 (52.3)	5 (33.3)	18 (62.2)	0.03
MRI				
T2 lesion volume in mm^3^ (IQR; *n* = 39)	2.4 (4.4)	1.0 (3.7)	4.0 (4.0)	n.s.
Brain parenchyma fraction (*n* = 19)	75.9 (3.0)	76.9 (1.3)	75.3 (3.5)	n.s.
Cervical transversal area in mm^2^ (*n* = 18)	103.3 (5.5)	100.0 (4.7)	103 0.0 (5.7)	n.s.
Treatment, *n* (%)				
METs	25 (49.0)	15 (60.0)	10 (40.0)	
HETs	26 (51.0)	3 (11.5)	23 (88.5)	<0.001

EDSS, Expanded Disability Status Scale; OCGB, Oligoclonal G bands in cerebrospinal fluid; OCMB, Oligoclonal M bands in cerebrospinal fluid; sNfL, serum Neurofilament Light chain; Low-sNfL cohort, patients presenting at baseline sNfL levels less than 11.0 pg/mL; High-sNfL cohort, patients presenting at baseline sNfL levels equal or more than 11.0 pg/mL; METs, moderate efficacy disease modifying treatment (teriflunomide, dimethyl fumarate, glatiramer acetate, fingolimod and cladribine). HETs, High efficacy therapy disease modifying treatment (natalizumab, anti-CD20 monoclonal antibodies, alemtuzumab and autologous stem cell transplant). n.s, not significant.

### ROC analysis

ROC analysis showed that a sNfL level equal or higher than 11 pg/mL predicts a EDSS equal or higher than 2 by the end of year 3 in patients with no AI, with a sensitivity of 83%, specificity of 59%, positive predictive value of 65%, negative predictive value of 80%, accuracy of 71%. The area under the curve of the test was 0.74. Further details in Supplementary Figure 1 and Supplementary Table 2.

### Study cohorts

**Figure 2:**
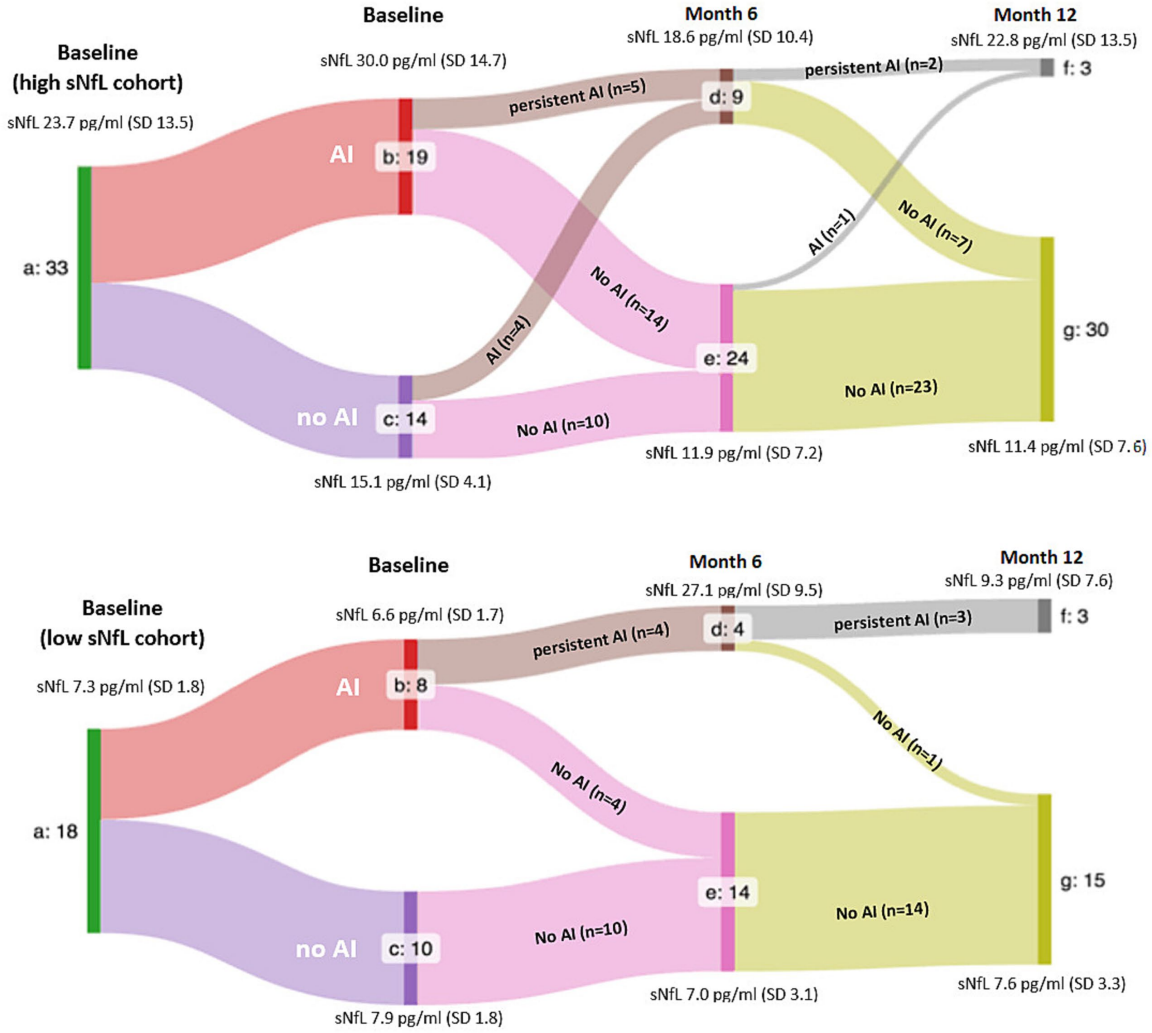
Flowchart of serum neurofilament light chain levels in the high and low serum neurofilament light chain cohorts stratified upon the presence of inflammatory activity at each time point. a: whole cohort. b, d, and f: patients presenting signs of acute inflammation (AI), c, e, and g: patients without signs of AI. Mean sNfL levels are shown. SD: standard deviation. Mean sNfL levels are shown. SD: standard deviation. *p*-values after stratification by the presence of AI were *p* < 0.001 at baseline, *p* = 0.006 at month 6 and *p* = 0.007 at month 12 in the high sNfL cohort, and not significant at baseline, p < 0.001 at month 6, and not significant at month 12 in the low sNfL cohort.

Patients with baseline sNfL levels of 11.0 pg/mL or higher defined the h-sNfL cohort (*n* = 33), while those with baseline levels below defined the l-sNfL cohort (*n* = 18). Figure 2 show flow diagrams of each cohort, respectively, showing the relative number of patients experiencing AI and their sNfL levels at each time point. At baseline, patients in the h-sNfL cohort exhibited features associated with higher inflammatory activity such as higher EDSS, increased proportion of HETs usage (88.5% vs. 11.5%), presence of OCMB in CSF (62.2% vs. 33.3%) and higher T2 lesion volume (T2LV) when compared to patients with l-sNfL. However, no significant differences among MRI parameters (T2LV, brain parenchyma, C2 transversal area) were detected. Further details are shown in Table 1. To explore the influence of time elapsed of disease onset to sNfL testing, Spearman’s correlations with sNfL levels and EDSS were performed, and none of them were significant (*p*-values 0.45 and 0.26, respectively).

**Figure 3:**
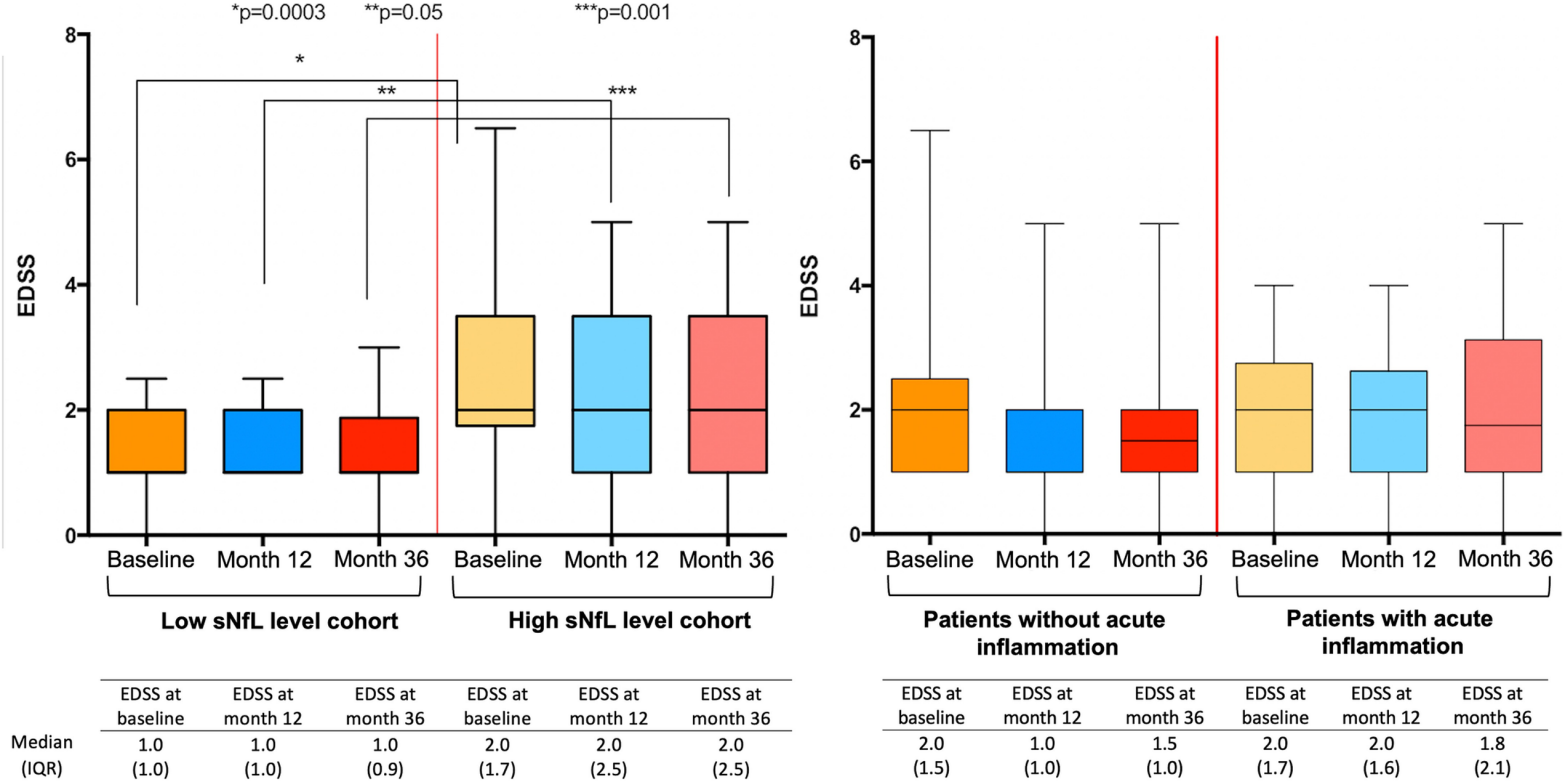
Expanded Disability Status Scale (EDSS) at every time point after stratification by baseline serum neurofilament light chain (sNfL) levels, and by the presence of acute inflammation (AI). Differences in EDSS between patients with and without AI were not significant at any time point.

With respect to the presence of AI, a higher proportion of patients in the h-sNfL cohort presented AI at baseline (57.5% vs. 44.4%) and at month 6 (57.5% vs. 44.4%). However, by the end of month 12, the opposite occurred (9.1% vs. 22.2%). In addition, the presence of AI was associated in the whole, l-sNfL and h-sNfL cohorts, with persistently higher mean sNfL levels at baseline, month 6 and 12. These differences were significant except for the l-sNfL cohort at month 6 (Figures 1–3).

Among the 8 patients who had sNfL levels below 11.0 pg/mL and concomitant AI at baseline, 4 were due to spinal cord relapses, 2 due to optic neuritis, and the other 2 due to brainstem syndrome. Further details in Supplementary Table 1.

sNfL levels of patients without signs of AI by the end of year 1 were still higher in the h-sNfL (11.4 pg/mL) than in the l-sNfL cohort (7.6 pg/mL). Also, disability of patients in the h-sNfL cohort were significantly higher throughout all time points, including at the end of year 3 (EDSS 2.0 [IQR 2.5] vs. 1.0 [IQR 0.9]; *p* = 0.001). In contrast, no differences were significant when patients were classified upon the presence of AI (Figure 3).

## Discussion

In this prospective study, we observed that baseline sNfL levels above 11 pg/mL were associated with a higher EDSS at baseline and at the end of year 3, regardless of relapses or radiological activity, and despite treatment with HETs. It is important to highlight that the mean time from symptom onset of the first relapse to baseline sNfL determination was barely 6 months, before the effect of any DMT. This provides further evidence to the value of sNfL as a biomarker of disability from very early in the disease course.

Traditionally, sNfL levels have been related to the presence of relapses and MRI activity, which we have defined as acute inflammation (AI). High sNfL levels have shown to correlate strongly with both concurrent and future AI, and this may contribute for the worse outcomes in these patients (23). In fact, our cohort with higher sNfL levels exhibited features at baseline consistent with more active MS, such as higher proportion of AI, OCMB and higher EDSS. However, the proportion of patients with AI by the end of the first year was reduced to a lesser extent in those with higher sNfL levels compared to those with lower sNfL levels (9.1% vs. 22.2%). Although this may appear paradoxical, most patients with high sNfL levels exhibited baseline features consistent with aggressive MS, which biased treatment toward HETs in almost 90% of these patients, resulting in better control of AI. Nevertheless, patients who initially had higher sNfL levels continued to exhibit elevated levels throughout the follow-up, even in the absence of signs of AI and despite being treated with HETs. It raises the question of whether elevation of sNfL in these patients may be attributable to the presence of subclinical inflammation, although we cannot completely discard the presence of radiological activity at time points between MRI scans.

We also observed that elevated sNfL levels by the end of the first year in patients not presenting AI, after the full effect of DMT can be presumed, were associated with a higher EDSS by the end of year 3, as depicted by the ROC analysis. However, we could not see an increase in EDSS during the follow-up period in either cohort, which is in line with studies previously mentioned in the manuscript. This could be attributed to short observation time, as several observational studies show that baseline sNfL levels above the 10 pg/mL threshold predict increased disability and progression over the following 6 years and more (14, 24, 25). Additionally, HET usage may have influenced these results, as nearly 90% of patients in the high sNfL cohort started HET, and the remainder were switched to HET as soon they experienced AI, in accordance with the standard of care.

Progression of disability that is not attributable to the presence of AI (ie. PIRA and PIRMA) is of particular concern, as this is the main driver of long-term disability in patients (4). The possibility that elevated sNfL levels unveil ongoing chronic inflammation and axonal destruction outside of AI is supported by studies involving progressive patients and long observation periods. These studies show that sNfL levels above the 10 to 11 pg/mL range are observed in patients experiencing progression compared to those with stable RRMS, and that these levels predict long-term progression (15, 26). Moreover, an observational study showed that increasing the cut-off to 15.6 pg/mL discriminates between benign and aggressive disease courses after more than 17 years of follow-up (27). Interestingly, our patients with sNfL levels above 11 pg/mL had twice the chance of having OCMB in the CSF, a biomarker associated with poor prognosis and early progression (18, 25). Hence, future studies with longer follow-up periods that consider DMT effect are needed to clarify the prognostic value of sNfL to detect future worsening in the new treatment era.

It is also remarkable that high sNfL levels, but not the presence of AI, was associated with increased disability. These are relevant findings, as it may indicate that AI may be indeed well controlled with current DMTs, especially HETs. However, the underlying mechanisms driving disability and maintaining persistently elevated sNfL levels are scarcely modified (6, 28).

On the contrary, patients with low baseline sNfL levels but concomitant AI experienced relapses restricted to the optic nerve, brainstem, or spinal cord. The latter observation aligns with previous findings suggesting that acute spinal cord lesions may not elevate sNfL levels (29). Thus, caution must be taken when interpreting sNfL levels, as they may not reflect accurately spinal cord damage, which has important prognostic implications.

Yet, sNfL levels have demonstrated to be a robust early biomarker for predicting increased long-term disability. Since early use of HETs has shown to prevent disability in the long term (30), sNfL could serve to assist in selecting patients with a favorable benefit-to-risk ratio for early treatment with these therapies.

Several limitations apply to this study in addition to those previously the aforementioned, such as the small sample size, and the use of absolute sNfL levels instead of age-adjusted levels (ie. Z-scores). However, the influence of age is mitigated by the fact that the curve of sNfL levels almost plateaus before the age of 40, which corresponds with the mean age of our cohorts (31). In addition, factors such as body mass index, renal function, subclinical systemic infections and other co-morbidities, known confounders of sNfL levels, were not considered.

These limitations preclude applying these results on a broader MS population and highlight the need for new studies that adjust for these factors. Finally, the presence of AI could be overlooked in patients experiencing Gd enhancement between MRI scans.

To conclude, patients with sNfL levels above 11.0 pg/mL at presentation, in the absence of classical signs of acute inflammation (i.e., MRI activity or relapses), maintain elevated levels despite receiving HETs and tend to have higher EDSS scores. We suggest that elevated sNfL in these patients may unveil mechanisms associated with early axonal degeneration that could aid to identify patients with higher risk of progression and should be considered in future trials.

## Data Availability

The raw data supporting the conclusions of this article will be made available by the authors, without undue reservation.
