# Supplementary material for: Clinical trajectories of patients with multiple sclerosis from onset and their relationship with serum neurofilament light chain levels
Source: Front Neurol. 2024 Oct 30;15:1477335. doi: 10.3389/fneur.2024.1477335 (PMC11559265; doi:10.3389/fneur.2024.1477335)
Supplement: Supplementary file 1 [file Table_1.DOCX]

| **Sex** | **Age at presentation (years)** | **Type of activity** | **Clinical presentation** | **sNfL level (pg/mL)** | **Time from relapse to sNfL determination (days)** |
| --- | --- | --- | --- | --- | --- |
| M | 24 | CR;  1 SC GEL | Spinal Cord | 3.09 | 12 |
| F | 29 | CR:  1 Brain GEL | Spinal Cord | 5.63 | 17 |
| M | 35 | CR:  No GEL | Spinal Cord | 6.25 | 14 |
| M | 29 | CR:  1 Brain GEL | Diplopia | 7.30 | 8 |
| F | 30 | CR:  1SC-GEL | Spinal cord | 6.85 | 2 |
| F | 42 | CR:  1 SC-GEL | Retrobulbar optic neuritis | 7.00 | 51 |
| M | 28 | CR:  1 Brian-GEL | Facial palsy | 8.39 | 71 |
| M | 26 | CR:  No GEL | Retrobulbar optic neuritis | 8.64 | 60 |

**Supplementary table 1**: Description of the 8 cases with acute inflammation ad concomitant levels of sNfL below 11.0 pg/ml.

CR: Clinical Relapses; SC: Spinal Cord; GEL: Gadolinium Enhancing Lesion; sNfL: serum Neurofilament Light Chain. M: Male. F: Female.
