# Supplementary figures and images for: Clinical trajectories of patients with multiple sclerosis from onset and their relationship with serum neurofilament light chain levels
Source: Front Neurol. 2024 Oct 30;15:1477335. doi: 10.3389/fneur.2024.1477335 (PMC11559265; doi:10.3389/fneur.2024.1477335)

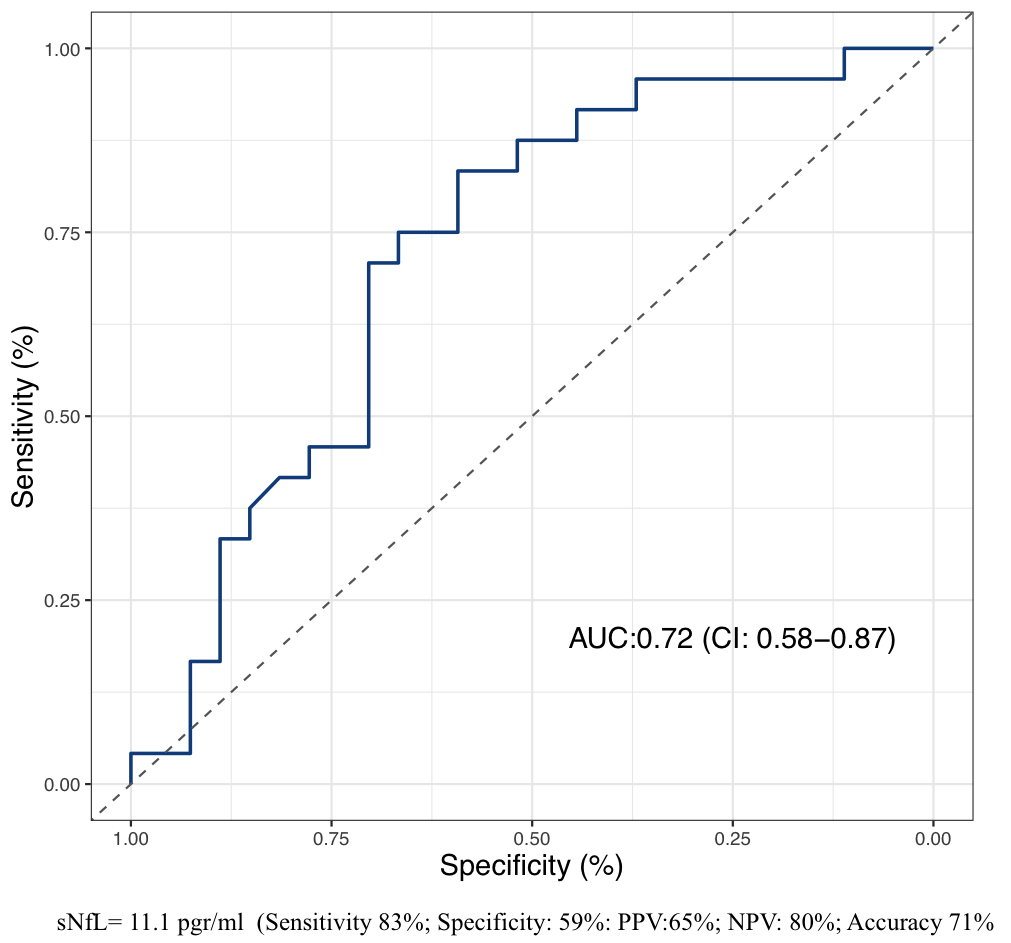

Supplement: Supplementary file 3 [file Image_1.PNG]

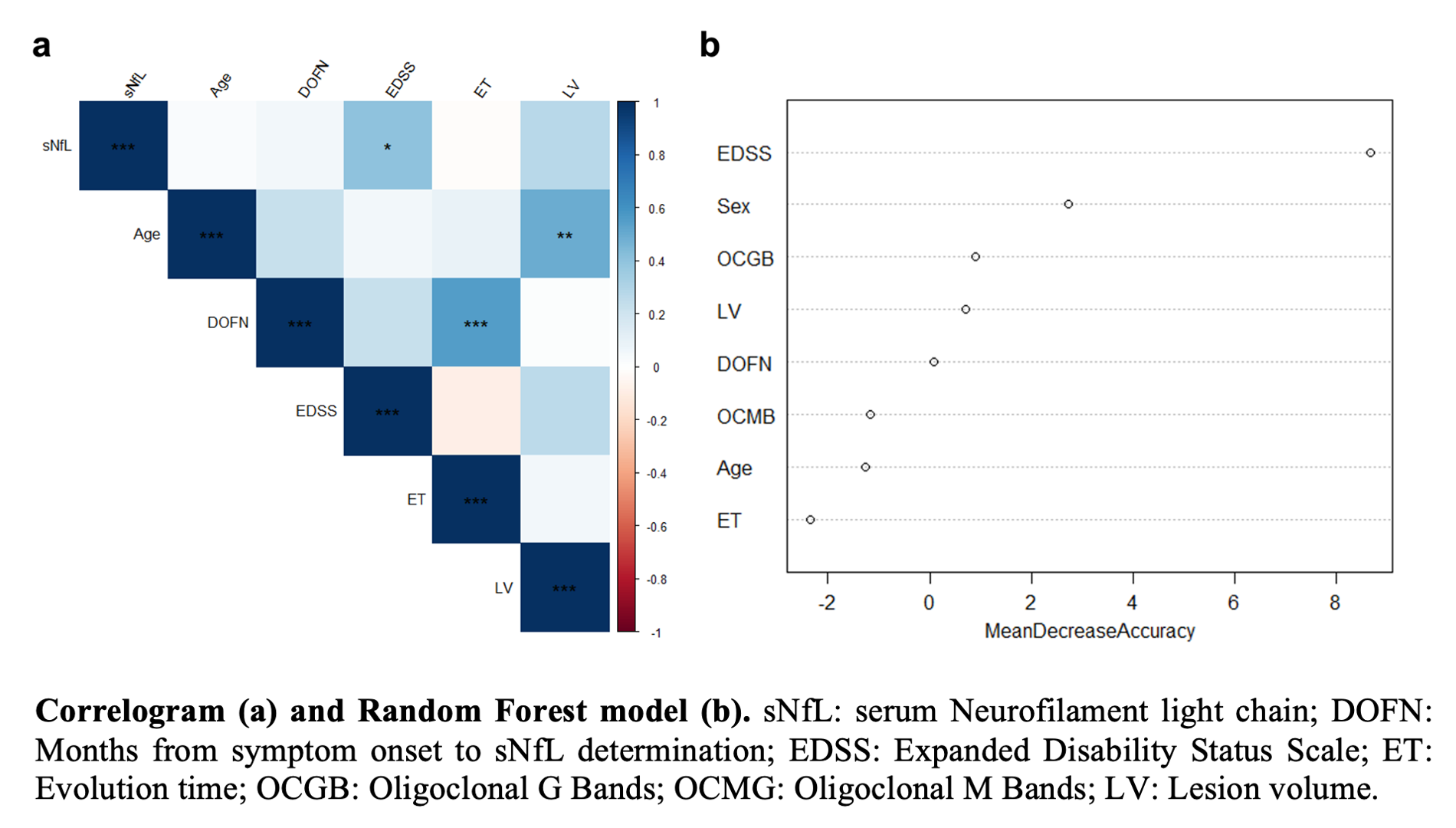

Supplement: Supplementary file 4 [file Image_2.PNG]
